# Annotating Argument Schemes

**DOI:** 10.1007/s10503-020-09519-x

**Published:** 2020-05-07

**Authors:** Jacky Visser, John Lawrence, Chris Reed, Jean Wagemans, Douglas Walton

**Affiliations:** 1 Centre for Argument Technology, University of Dundee, Nethergate, Dundee, DD1 4HN UK; 2 Amsterdam Centre for Language and Communication (ACLC), University of Amsterdam, Spuistraat 134, Amsterdam, 1012 VB The Netherlands; 3 Centre for Research in Reasoning, Argumentation and Rhetoric, University of Windsor, 401 Sunset Avenue, Windsor, ON N9B 3P4 Canada

**Keywords:** Annotation, Argument scheme, Argumentation scheme, Argument scheme key (ASK), Argument Type Identification Procedure (ATIP), Corpus, Election debates, Periodic table of arguments

## Abstract

Argument schemes are abstractions substantiating the inferential connection between premise(s) and conclusion in argumentative communication. Identifying such conventional patterns of reasoning is essential to the interpretation and evaluation of argumentation. Whether studying argumentation from a theory-driven or data-driven perspective, insight into the actual use of argumentation in communicative practice is essential. Large and reliably annotated corpora of argumentative discourse to quantitatively provide such insight are few and far between. This is all the more true for argument scheme corpora, which tend to suffer from a combination of limited size, poor validation, and the use of ad hoc restricted typologies. In the current paper, we describe the annotation of schemes on the basis of two distinct classifications: Walton’s taxonomy of argument schemes, and Wagemans’ Periodic Table of Arguments. We describe the annotation procedure for each, and the quantitative characteristics of the resulting annotated text corpora. In doing so, we extend the annotation of the preexisting US2016 corpus of televised election debates, resulting in, to the best of our knowledge, the two largest consistently annotated corpora of schemes in argumentative dialogue publicly available. Based on evaluation in terms of inter-annotator agreement, we propose further improvements to the guidelines for annotating schemes: the argument scheme key, and the Argument Type Identification Procedure.

## Introduction

Theory-driven and data-driven studies of argumentation alike rely on data about the actual use of argumentation in communicative practice—to test top-down theorising, or found a bottom-up empirical approach. This data can come from the qualitative appraisal of selected examples, or from quantitative approaches. While labour intensive, the latter are gaining traction—motivated in part by the requirements of machine learning methods for automated text processing. Quantitative approaches require (preferably large) corpora of actual argumentative discourse annotated with the necessary theoretical notions and concepts.

In the current paper, we address the annotation of text corpora with argument schemes. To elucidate the general approach, specific procedures, and value of the outcomes, we make use of two distinct typologies representing different theoretical perspectives and rationales. Douglas Walton’s taxonomy of argumentation schemes (Sect. 3.1) is an empirically oriented classification of schemes based on the examination of the apparent conventions of argumentative practice. Jean Wagemans’ Periodic Table of Arguments (Sect. 3.2) is at the other end of the spectrum, starting from multiple a priori criteria devised to exhaustively describe all possible instantiations in actual practice. In the current paper, we do not wish to evaluate the appropriateness or correctness of any theory—whether Walton’s or Wagemans’ or any alternative. Like many alternatives, both approaches have their place and value within scholarly traditions and for various applications: Walton’s taxonomy has found wide uptake in the study of argumentation (within both traditional and computational approaches), while Wagemans’ decompositional approach is applied in formal linguistics and yields possible advantages for automation and explication—and other alternatives have their own advantages and disadvantages.

Instead of reflecting on the theoretical or practical validation of any particular approach, we focus on the annotation of a corpus on the basis of the two distinct typologies. In doing so, we aim to explain the annotation task and its difficulties (Sect. 2), draw annotation guidelines from the two typologies (Sect. 3), present the resulting annotated corpora (Sect. 4), suggest ways of improving the annotation of schemes (Sect. 5), and illustrate two applications of the resulting corpus data (Sect. 6).

## Argument Scheme Corpora

### Conceptions of Argument Schemes

Understanding the inferential principles underpinning argumentation is essential to its proper interpretation and evaluation. Since antiquity, explicating these inferential principles has been one of the main scholarly objectives in the study of argumentation (Rubinelli 2009). As one such explanation, the notion of ‘argument(ation) scheme’ was introduced during the second half of the 20th century (Garssen 2001). Although Perelman and Olbrechts-Tyteca (1969) introduced the similar notion of ‘argumentative scheme’ in *The New Rhetoric*, the current understanding of argument scheme goes back to Hastings’ (1963) PhD thesis and the conceptualisation in van Eemeren et al. (1978)’s first handbook on *Argumentation Theory*.^1^ Argument schemes capture the conventionally acceptable patterns of reasoning that are appealed to in argumentative communication, substantiating the inferential connection between premise(s) and conclusion. The defeasibility of the schemes sets them apart from the strict reasoning patterns of classical formal logic (e.g., *modus ponens*), as does the dialogical nature of the schemes evident in their association with ‘critical questions’ used to evaluate the acceptability of an applied argument(ation) scheme.^2^

Since their introduction, argument schemes have become a central issue in modern argumentation studies, leading to a variety of classifications, e.g., by Schellens (1985), Kienpointner (1992), van Eemeren and Grootendorst (1992), and van Eemeren and Garssen (2019). Although the concept was developed for different purposes, it has also found uptake in computer science and artificial intelligence (Rahwan and Simari 2009; van Eemeren et al. 2014; Baroni et al. 2018). Within these areas, Walton’s approach to argument schemes (Walton 1996; Walton et al. 2008) is most influential (van Eemeren et al. 2014). Walton’s classification comprises a great variety of schemes, described in some detail, but with the flexibility to allow adjustments in order to fit a scheme to a desired domain-specific application or research project [see, e.g., the revisions and extensions of the *practical reasoning* scheme by Atkinson and Bench-Capon (2018), and Kokciyan et al. (2018).]

One of the great challenges facing this work has been the principled organisation and taxonomisation of schemes (see, e.g., Katzav and Reed (2004); Walton and Macagno (2015)), leading other authors such as Wagemans (2016) to propose a priori exhaustive grounds for scheme definition and classification. While Walton’s classification can be characterised as an empirically motivated taxonomy of types of argument encountered in argumentative practice, Wagemans’ Periodic Table of Arguments is a factorial typology that specifies a comprehensive set of argument types on the basis of a limited set of theoretical descriptions of argument characteristics.

### Existing Argument Scheme Annotations

The development of large-scale data resources consisting of text (corpora) in which certain characteristics have been annotated with codes expressing particular argumentative notions and concepts constitutes a corpus-linguistic approach to argumentation: the collection and annotation of a textual dataset (‘corpus’) to aid the quantitative empirical study of language (‘linguistics’). The notions and concepts used for annotation are ordinarily based on a particular theoretical framework. To ensure the annotation is reliable and consistent, explicit guidelines are specified, containing instructions for annotators.

Existing text corpora annotated with argument schemes tend to be based on Walton’s taxonomy, although some alternatives are explored. A constant, however, is that evaluations of the reliability of the annotation methods show that this is a difficult task to perform. Lindahl et al. (2019), for example, annotated a corpus of Swedish newspaper editorials, achieving low agreement between the annotators. Musi et al. (2016) present a set of annotation guidelines on the basis of the Argumentum Model of Topics (Rigotti and Greco 2019), achieving a Fleiss’ (1971) $\kappa$ of 0.1 with minimally trained non-expert annotators, increasing to 0.307 after further training with improved guidelines.

For quantitative studies and for computational machine learning applications alike, it is important that annotated corpora are sizeable and balanced, reliably distinguishing a broad range of scheme types. However, many existing argument scheme annotation projects start from a limited ad hoc selection of scheme types to take into consideration. For example, Duschl (2007) initially adopts a selection of nine argument schemes described by Walton (1996), for his annotation of transcribed middle school student interviews about science fair projects. At some point during the study, however, he collapses some of the classes to end up with four more general coding labels no longer directly related to any particular scheme types. This deviation from Walton’s theoretically motivated taxonomy appears to be only motivated by the need to improve annotation agreement. The validation of the annotation method does not account for chance agreement, by only providing percentage-agreement scores (instead of resorting to, e.g., Cohen’s (1960) $\kappa$). Out of a total of 17 annotated texts, Duschl (2007) reports the inter-annotator agreement on two as being, respectively, 90% and 84%. No detail is provided on the sampling method.

In a similar way, Song et al. (2014) base their annotation on a modification of Walton’s taxonomy, settling on a restricted set of three more general schemes: *policy*, *causal*, and *sample*—resulting in Cohen’s $\kappa$ scores for inter-annotator agreement ranging widely from 0.364 to 0.848. Anthony and Kim (2015) employ a bespoke set of nine coding labels modified from the categories used by Duschl (2007) and nine schemes described in a textbook by Walton (2006). They do not measure any inter-annotator agreement, opting for a fully open collaborative annotation without any testing of reliability of the methods. Cabrio et al. (2013) explore correlations between the Penn Discourse Treebank (Prasad et al. 2008) and a selection of five schemes from those presented by Walton (1996)—*argument from example*, *argument from cause to effect*, *argument from effect to cause*, *practical reasoning*, and *argument from inconsistency*—while also suggesting two new ones: *argument from equivalence* and *argument from specification*.

### Argument Scheme Annotation Task

#### Source Data

The source data in our argument scheme annotation task comprises transcripts of televised debates for the 2016 presidential elections in the United States of America. The communicative context in which the debates takes place influences the argumentative activity, as it determines, e.g., the outcomes aimed for, the roles of the participants involved, and the rules or conventions with respect to the argumentative means available to them (van Eemeren 2010). The interests and values of the individual participants further shape the practice (Fairclough 2006): the context of televised election debates is heavily influenced by the candidates’ objective to persuade the electorate to vote for them, and the broadcasting networks’ aim of providing a fair and well-viewed platform for doing so.

Ever since the first televised election debate between then US presidential candidates John F. Kennedy and Richard Nixon in 1960, television debates have played an important role in the democratic process in many countries (Kraus 2013). The general election and the associated television debates between Hillary Clinton and Donald Trump as the candidates for the two dominant political parties in the US (respectively the Democratic Party and the Republican Party) took place in the Autumn of 2016. Prior to the general elections, both main parties held primary elections and caucuses to elect their party’s candidate for the presidency. These primaries were also preceded by television debates between the leading prospective candidates in 2015 and 2016.

While the format of each of the debates is slightly different, there are some recurring characteristics. Being television debates, the discourse is spoken, with transcripts available retrospectively through a variety of sources, and video recordings broadcast live and available afterwards. The participants are expected to use language that is appropriate for the occasion: balancing the political nature of the issues discussed with the need to keep the proceedings comprehensible for a broad untrained audience. A selection of a limited number of candidates is invited to these events, moderated by anchors and journalists from the television networks that air them (among others, CBS, CNN, Fox News, and NBC). The television networks’ moderators pose questions to the invited candidates, and guide the debate (for example by keeping time and order), while the candidates make opening statements, answer the moderators’ (and occasionally the public’s) questions, defend their views and challenge those of their political opponents, in an attempt to garner more support among the electorate.

For the primaries, the Republican party held 12 debates for the front-runners and seven so-called ‘undercard’ debates between the next tier of candidates. The Democratic party held 10 primary debates. As time went on and more of the candidates withdrew their candidacy, the number of participants declined over the course of these series of debates. For the general elections, three television debates were organised between Democratic candidate Clinton and Republican candidate Trump, and one debate between their vice-presidential candidates.

The argumentation encountered in the debates is not always explicitly signalled with linguistic markers, nor necessarily cohesive. The television debates are a spoken genre of discourse. This means that the history of the dialogue is not entirely available to the participants, as they forget details of what transpired in the earlier stages of the debate. This may lead to occasional repetitions and contradictions of what was said earlier. Furthermore, candidates cannot always rely on their prepared and practised lines and topics, but have to respond to unexpected turns and twists, and to interaction with the other candidates and moderators. Because responding well to such dynamic situations is expected to instil the voters’ confidence in the candidate, candidates receive support to varying degrees from communication professionals in their preparation and training, and rely on their experience in political debate.

The context of televised election debates fosters a mixture of well-structured and well-presented argumentation that appears to have been prepared in advance, and impromptu argumentation originating from the need to cope with the interactional dynamics. The level of noise in the data—in terms of e.g. crosstalk, unconventional use of discourse markers, and low discourse cohesion—poses a challenge in the analysis of the argumentation. Consider Example (1), advanced by then prospective candidate Trump. Trump anticipates that his claim about the topic of immigration will not be outright accepted. He therefore supports it with multiple statements, but does so in a non-straightforward fashion. Upon closer inspection, Trump’s support relies mostly on the rhetorical device of repetition, with several of his assertions constituting rephrases instead of inferences.

(1): Donald Trump: *So, if it weren’t for me, you wouldn’t even be talking about illegal immigration, Chris. You wouldn’t even be talking about it. This was not a subject that was on anybody’s mind until I brought it up at my announcement. And I said, Mexico is sending. Except the reporters, because they’re a very dishonest lot, generally speaking, in the world of politics, they didn’t cover my statement the way I said it.*

#### The US2016 Corpus

The US2016 corpus presented by Visser et al. (2019a) contains annotated transcripts of the first television debates leading up to the primaries of the Democratic party, the primaries of the Republican party, and the 2016 General Election for the US presidency (Peters and Woolley 1999). Additionally, the US2016 corpus contains related annotated social media posts extracted from the Reddit social media platform (http://www.reddit.com), and the argumentative interaction between the user-generated social media posts and the candidates’ debating on television (Visser et al. 2018a). The collected corpus was annotated by four annotators on the basis of Inference Anchoring Theory (IAT) (Reed and Budzynska 2011). Building on insights from discourse analysis and argumentation studies, IAT conceives of argumentative conduct in terms of the anchoring of argumentative reasoning in communicative interaction. By reinterpreting the speech act theoretical notion of ‘illocutionary force’ (Austin 1962; Searle 1969), the eponymous anchoring is theoretically conceptualised as the intentional ‘illocutionary connection’ between argumentative content and locutions in dialogue. IAT annotations capture the propositional structure of argumentation as well as its dialogical structure, in a machine-readable format to facilitate computational processing (Chesñevar et al. 2006).

The full version of the IAT annotation guidelines is available online at http://arg.tech/US2016-guidelines. Here we reproduce a summary selection of the essential notions.

- *Segments* divide the (transcribed) text into locutions, consisting of a speaker designation and an ‘argumentative discourse unit’ (a text span with discrete argumentative function) (Peldszus and Stede 2013).
- *Transitions* capture the functional relationships between locutions, reflecting the dialogue protocol—a high level specification of the set of transition types that are available in a particular communicative activity.
- *Illocutionary connections* embody the intentional communicative functions of locutions or transitions, such as: *Agreeing*, *Arguing*, *Asserting*, *Challenging*, *Disagreeing*, *Questioning*. Some types of illocutionary connection lead to the reconstruction of a propositional content.
- *Inferences* are directed relations between propositions, reflecting that a proposition is meant to supply a reason for accepting another proposition. An argument scheme can be specified; failing that, it is labelled as *Default Inference*.

The full annotation guidelines have been validated by calculating the inter-annotator agreement on a 11.3% sample, resulting in a Cohen (1960)’s $\kappa$ of 0.610, and a CASS (Duthie et al. 2016) $\kappa$ of 0.752—both indicating substantial agreement according to Landis and Koch (1977)’s standard interpretation of the kappa metric. The resulting annotated US2016 corpus is freely available online at http://corpora.aifdb.org/US2016. Table 1 compiles representative quantitative characteristics of the US2016 corpus: word count, and counts of, e.g., text segments (‘locutions’), arguments (‘inference’), counterarguments (‘conflict’), and the most relevant types of illocutionary connections (Visser et al. 2019a).

In addition to the characteristics of the US2016 corpus, Table 1 shows the properties of the US2016G1tv sub-corpus comprising the annotated transcript of the first head-to-head debate for the general elections between Clinton and Trump (Peters and Woolley 2016). We include this sub-corpus in particular, because it constitutes the source material for our argument scheme annotation.

**Table 1:** Properties of the US2016 and US2016G1tv corpora

Corpus	Word tokens	Locutions	Illocutions	Propositions	Inference	Conflict	Rephrase	Arguing	Disagreeing	Restating
US2016	97,999	8937	13,331	8099	2830	942	764	788	907	576
US2016G1tv	17190	1584	2285	1473	505	79	140	507	62	121

#### Annotation Procedure

The IAT annotation of the US2016G1tv corpus resulted in 505 inferential argumentative relations. Each of these relations can be classified as instantiating a particular reasoning principle—its argument scheme. For example, during the first general election debate, Clinton advanced the argument in Example (2).

(2): Hillary Clinton: *And we finally need to pass a prohibition on anyone who’s on the terrorist watch list from being able to buy a gun in our country. If you’re too dangerous to fly, you are too dangerous to buy a gun.*

**Fig. 1:**
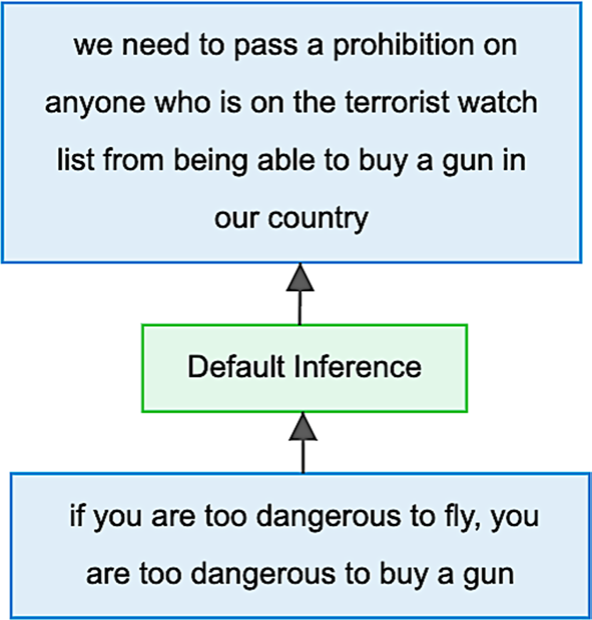
Diagrammatic visualisation of Example (2) in the IAT-annotated US2016G1tv corpus

Example (2) is annotated with IAT in the US2016G1tv corpus as two propositions connected by a directed inference relation from premise to conclusion. As visualised in Fig. 1, the inference relation between the two propositions is initially left unclassified—the ‘Default Inference’ node connects the premise at the bottom to the conclusion at the top. The task of annotating argument schemes consists of classifying these inferential relations on the basis of a particular typology—in our case, with Walton’s taxonomy of argumentation schemes, and Wagemans’ Periodic Table of Arguments.

Two annotators trained in argumentation analysis and with prior knowledge of argument schemes used the annotation guidelines laid out in Sects. 3.1.2 and 3.2.2 to classify 55% of the inferential relations in the US2016G1tv corpus. These new annotations form an extension of the preexisting US2016G1tv corpus, resulting in two new corpora that contain the original IAT annotation and the classifications of the argument schemes (see Sect. 4). The random allocation of 55% of the 505 inferences in the corpus to the two annotators results in a 10% overlap. This random sample is used for calculating the reliability of the guidelines (see Sect. 5).

## Annotation Guidelines for Argument Schemes

### Annotation with Douglas Walton’s Taxonomy

#### Taxonomy of Argumentation Schemes

The known set of argumentation schemes should not be regarded as complete or immutable, but rather as a work in progress that is continually subject to readjustment and refinement as concepts defining the schemes are formulated in more precise ways and applied to new examples. Improving the classification system of schemes is a continuous process of adjustment between collecting data, sharpening criteria that enable the identification of a scheme, and used to refine the taxonomy to assist the ongoing collection of data.

The classification system byWalton et al. (2008, pp. 349–350) divides schemes into three general categories: reasoning, source-based arguments, and applying rules to cases. Under reasoning five subcategories are distinguished: deductive reasoning, inductive reasoning, practical reasoning, and abductive reasoning. Under the general heading of source-based arguments, four schemes are listed: *argument from position to know*, *argument from commitment*, *argument attacking personal credibility*, and *argument from popular acceptance*. The third general category is called ‘applying rules to cases’. It has four subcategories: arguments based on cases, defeasible rule-based arguments, verbal classification arguments, and chained arguments connecting rules in cases. Each of these second-level types of schemes contains categories at a finer level of granularity. These third-level schemes include many of the schemes that are so highly familiar to researchers on argumentation. For example, the third category under source-based arguments contains the following three schemes: *argument from allegation of bias*, *poisoning the well by alleging group bias*, and *ad hominem* arguments.

Walton et al. (2008, p. 348) acknowledge that because of the difficulty of defining the concepts that any classification system of schemes has to be based on—such concepts as knowledge, causation, threat, and so forth—any attempt to classify schemes faces conceptual difficulties in adequately defining the contested concepts used at the top levels of the tree structure. For this reason, the 2008 system of classifying schemes is to be regarded as a provisional hypothesis that should be subject to improvement as further empirical and analytical work on schemes classification continues. In the ten year interval, the explosion of research on argument mining (Lawrence and Reed 2019; Stede and Schneider 2018) has raised many fine-grained questions about how particular groups of schemes should be fitted together into the larger picture of any general classification system.

Subsequent work by Walton and Macagno (2015) presents a survey of the literature on scheme classification, as well as outlining how the 2008 system can be modified in order to accommodate current research in artificial intelligence and computational linguistics on argument mining. It was shown how the procedure of developing and using classification systems can only move forward by combining two approaches. One of these is a top-down approach that begins with concepts formulated at a high level of abstraction that any classification system of schemes has to be based on, including such concepts as knowledge, causation, threat, and so forth. This top-down approach then moves to particular types of schemes that fit under these general categories. Finally, it moves to schemes representing the types of arguments we are already so widely familiar with. But at the same time, as progress on argument mining and other quantitative empirical research continues, it is also necessary to have a bottom-up approach that begins with real examples of arguments at the ground level of cases that distinguish in a very particular way between subtypes of a given scheme (Walton 2012). What happens at this bottom-up level is that commonalities between patterns of reasoning are used to start to build clusters of schemes, and then these clusters have to be fitted into more general classifications of schemes, resulting in a classification system in which the higher levels have been developed a priori and the lower levels by empirical generalisations.

A central practical problem inherent in the current experimental work on argumentation schemes with corpus-linguistic and computational-linguistic approaches is that the annotators lack enough specific guidance on how to decide whether an argument found in a real natural language text can properly be said to fit a particular scheme or not. An early study on the annotation of kinds of arguments put forward by candidates in a provincial election in Canada classified 256 arguments using 14 schemes and a category called “none of the above” (Hansen and Walton 2013). A group of six coders, two of them experts in argumentation theory, found it difficult to classify arguments in some instances because of the open texture of key terms used in the schemes. As a solution to this problem, Walton (2012) recommends devising a set of identification conditions that can be used to offer coders additional guidance on whether a particular scheme fits a particular case or not. In Sect. 3.1, we build on these identification conditions to extend and refine our annotation guidelines, by functionally clustering the set of 60 upper level schemes catalogued by Walton et al. (Walton et al. 2008, pp. 308-346) for the practical task of text annotation.

#### Annotation Guidelines for Walton’s Taxonomy

Walton’s taxonomy of argumentation schemes is operationalised as annotation guidelines on the basis of the *Argumentation Schemes* book from 2008 (Walton et al. 2008). More specifically, the guidelines consist of Chapter 9 of the book: *A User’s Compendium of Schemes* (Walton et al. 2008, pp. 308–346), which comprises an extensive description of 60 main scheme types. The annotation classes are constrained to these 60 main schemes, disregarding the many listed variants. Examples not fitting any of the 60 schemes are labelled ‘default inference’ to indicate that they elude classification with Chapter 9 of the *Argumentation Schemes* book (following Hansen and Walton’s (2013) use of the ‘none of the above’ category). Since the annotation of argument schemes is an extension of the existing IAT annotation of the basic US2016G1tv corpus, in some cases, the scheme labels need to be applied in a simplified, condensed or partial manner, to fit the pre-existing structure.

**Fig. 2:**
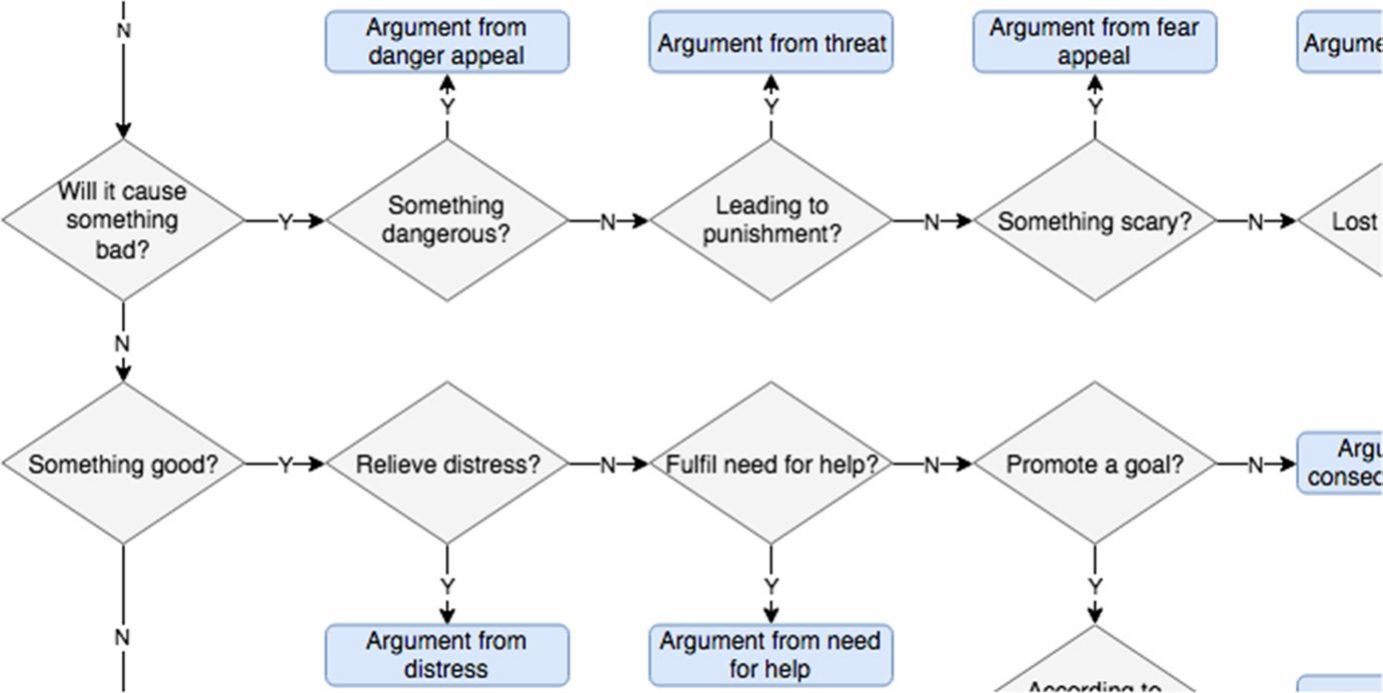
Fragment of the decision tree heuristic for distinguishing between action-oriented argument schemes in Walton’s taxonomy

To facilitate their decision making, the annotation guidelines are supplemented by a classification decision tree—a fragment of which is shown in Fig. 2. Primarily intended as an indicative annotation heuristic, the decision tree systematises the scheme set according to argument properties indicative of particular schemes. Each of the top-level branches of the tree represents divisions into general categories (for example, arguments based on character, or based on opinion), before breaking these down further by following a path of simple binary questions until a particular scheme classification is reached. In using the decision tree heuristic, the annotators follow the arrows of the flow chart, making yes/no choices at each diamond-shaped binary question, to eventually end up at an argument scheme, represented in the rounded squares, or the fallback *default inference* label if the argument does not fit any of the schemes in Walton’s (2008) taxonomy. The idea of an annotation decision tree will be further explored in Sect. 5.1.

### Annotation with Jean Wagemans’ Periodic Table of Arguments

#### Periodic Table of Arguments

A recently developed classification of argument types (Gobbo and Wagemans 2019a, b, c; Visser et al. 2018b; Wagemans 2016, 2017, 2018a, b, 2019a), the *Periodic Table of Arguments* (PTA) aims to provide a comprehensive and exhaustive alternative for the variety of existing taxonomies of argumentative techniques. The PTA presents a transparent theoretical rationale for distinguishing between the types of argument. It uses formal(isable) language for characterising these types, and it integrates the dialectical and rhetorical conceptualisations of argument into a single systematic and comprehensive whole.

The PTA conceives of an argument type as a characterisation of an inference relation, i.e., the specific way in which a premise supports a conclusion. The argument types distinguished within its theoretical framework are ‘atomic’ in the sense that they consist of one premise and one conclusion, both of which are expressed by means of a statement that consists of a subject and a predicate. Following logical conventions, subjects are indicated with lowercase letters *a*, *b*, etc., predicates with uppercase *X*, *Y*, etc. (predicate $\top$ having the fixed meaning ‘true’), and complete propositions with letters *p*, *q*, etc.

The theoretical framework of the PTA consists of three independent partial characterisations of argument, namely as (1) a first-order or second-order argument; (2) a predicate or a subject argument; (3) a specific combination of types of statements.^3^ The superposition of these three partial characterisations yields a factorial typology of argument that can be used in order to develop tools for analysing, evaluating, and producing arguments in natural language.

The distinction between *first-order* and *second-order* arguments hinges on the complexity of the statements that function as the premise and the conclusion of the argument. First-order arguments contain simple statements that cannot be broken down any further—such as ‘The suspect was driving fast, because he left a long trace of rubber on the road’. Second-order arguments are different in that they contain at least one complex statement, the subject of which can be broken down into a subject and a predicate itself. An example is ‘We only use 10% of our brain, because that was said by Einstein’, which has the conclusion about brain use functioning as the subject of the premise.

The distinction between *predicate* and *subject* arguments draws on the differences and similarities between the constituents of the conclusion and the premise of the argument. In short, the statements of predicate arguments contain the same subject and different predicates, while those of subject arguments contain different subjects and the same predicate.

Finally, using a widely accepted tripartite typology of statements developed in debate theory, the conclusion and premise of arguments are characterised as statements of *fact* (*F*), statements of *value* (*V*), and statements of *policy* (*P*). By identifying the type of statement of the conclusion and the premise, arguments can be characterised as a specific combination of types of statements—for example, a *PF* argument combines a statement of policy in its conclusion and a factual statement in its premise.

When taken together, these three partial characterisations of argument constitute a theoretical framework that allows for 2 × 2 × 9 = 36 different types of argument. Their systematic names consist of indicators for the three partial characterisations mentioned above. The prefixes *1* and *2* indicate first-order and second-order arguments. The infixes *pre* and *sub* indicate predicate arguments and subject arguments. Finally, combinations of *P*, *V* and *F* as suffix distinguish the various combinations of statements of policy, value and fact, respectively.

The systematic names of the 36 types can subsequently be related to corresponding ‘trivial’ names known from the literature on argument schemes and related taxonomies, with each systematic type hosting an arbitrary number of trivially named ‘isotopes’. An argument that has been classified as a first-order predicate argument combining a statement of fact with another statement of fact, for instance, would be labelled with the systematic name *1 pre FF*. Depending on the linguistic expression of the relationship between the two factual statements, the trivial name of this argument could be *argument from sign*, *from cause*, *from effect*, or similar.

#### Annotation Guidelines for Wagemans’ Periodic Table of Arguments

Because the typology of the PTA is based on the interplay between three distinctive characteristics of the arguments, the annotation task has been decomposed into three partial classification sub-tasks. In one of the sub-tasks, each proposition is classified as being of one of the three types distinguished in the PTA. The remaining two sub-tasks pertain to the inference relations between the propositions.

A proposition is classified as a proposition of *fact* if its veracity can be verified through empirical observation. It is classified as a proposition of *value* if it contains some evaluation (such as, ethical (e.g. right/wrong), aesthetical (e.g. beautiful/ugly), legal (e.g. guilty/innocent), or logical (e.g. true/false) evaluations). It is classified as a proposition of *policy* if it expresses a plan of action or an act to be carried out.

An inference relation is classified as *first-order* if it connects two propositions each containing a subject-predicate pair. It is classified as *second-order* if its premise is a locution (often the result of reported speech), or if the premise is otherwise applying a predicate to the full proposition of the conclusion.

Finally, inference relations are classified as a *predicate* or a *subject* argument. The relation is classified as a *predicate* argument if the propositions involved share the same subject term to which different predicates are applied, and as a *subject* argument if vice versa.

## Annotation Results

### Results of Annotation with Walton’s Taxonomy

**Fig. 3:**
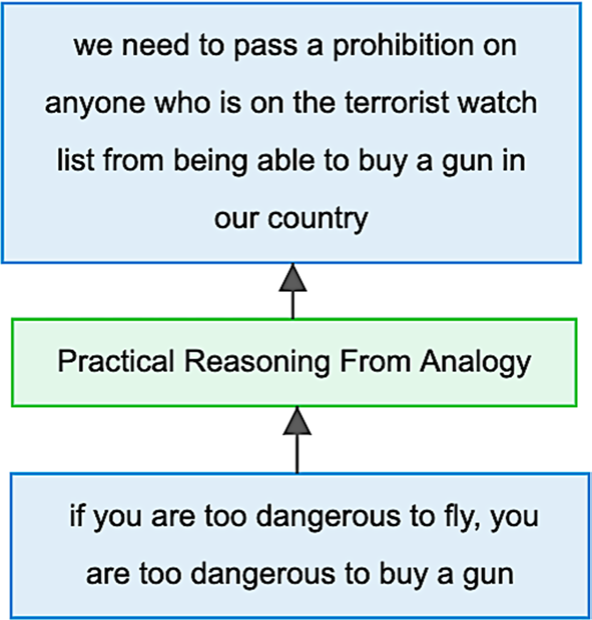
Diagrammatic visualisation of the annotation of Example (2) as an instance of *practical reasoning from analogy* in the US2016G1tvWALTON corpus

The results of the annotation in accordance with Walton’s classification of argumentation schemes are collected in the US2016G1tvWALTON corpus (freely available online at http://corpora.aifdb.org/US2016G1tvWALTON). In the corpus, the argument schemes are represented as part of an AIF-compliant graph structure (see Sect. 2.3.2), an example of which can be seen in Fig. 3. This figure shows the original IAT-segmented propositions of Example (2), and the inference relation between them classified as an instance of *practical reasoning from analogy*.

Of the 505 inference relations in the original US2016G1tv corpus, a total of 491 are annotated with one of the 60 argument scheme types in Walton’s classification, leaving only 14 as unclassified *default inference*. As the scheme counts in Table 2 show, the most common scheme, by some margin, is *argument from example*.

**Table 2:** Counts of argument schemes in the US1816G1tvWALTON corpus

Argument scheme	Count
Argument from example	81
Argument from cause to effect	48
Practical reasoning	45
Argument from consequences	40
Argument from sign	38
Argument from verbal classification	32
Generic ad hominem	28
Circumstantial ad hominem	24
Pragmatic argument from alternatives	23
Argument from values	15
*Default inference*	*14*
Argument from position to know	13
Argument from fear appeal	11
Argument from alternatives	9
Argument from bias	9
Argument from analogy	8
Argument from popular opinion	8
Argument from danger appeal	7
Argument from popular practice	7
Argument from composition	6
Ethotic argument	5
Practical reasoning from analogy	4
Argument from commitment	3
Argument from expert opinion	3
Argument from waste	3
Argument from gradualism	2
Argument from need for help	2
Argument from oppositions	2
Argument from perception	2
Argument from correlation to cause	1
Argument from definition to verbal classification	1
Argument from division	1
Argument from ignorance	1
Argument from rules	1
Argument from vagueness of verbal classification	1
Argument from witness testimony	1
Argumentation from interaction of act and person	1
Pragmatic inconsistency	1
Two-person practical reasoning	1

### Results of Annotation with the Periodic Table of Arguments

Table 3 collects the results of the three partial annotations on the basis of the PTA: *first*-/*second-order* and *predicate*/*subject* arguments, and propositions of *fact*, *value* and *policy*. The great majority of annotated arguments are *first-order*: 481 out of the total of 505. There are also more than twice as many *predicate* arguments as there are *subject* ones. Furthermore, propositions of *value* and *fact* greatly outnumber propositions of *policy*. Table 3 also lists the number of ‘unclassified’ results in each sub-task.

**Table 3:** Results of the partial annotation tasks based on the Periodic Table of Arguments

	Total number	First-order argument	Second-order argument	Unclassified	Subject argument	Predicate argument	Unclassified	Proposition of value	Proposition of policy	Proposition of fact	Unclassified
Inference relations	505	481	11	*13*	124	308	*73*	$\cdot$	$\cdot$	$\cdot$	$\cdot$
Related propositions	796	$\cdot$	$\cdot$	$\cdot$	$\cdot$	$\cdot$	$\cdot$	383	110	289	*14*

From these partial results, an aggregated final classification is derived, assigning to each of the argumentative inferences one of the 36 possible main types of the PTA (e.g. *1 pre FF*). If the partial classification of any of the inference relations or propositions involved in an argument failed (‘unclassified’ in Table 3), this leads to a classification as *Default Inference* in the final aggregation step. Similarly, any inference relation involving several premises without a dominant proposition type is assigned *Default Inference*. The final classification is included in the US2016G1tvWAGEMANS corpus (freely available online at http://corpora.aifdb.org/US2016G1tvWAGEMANS).

**Table 4:** Counts of argument types in the US2016G1tvWAGEMANS corpus

Argument type	Count
Default inference	85
1 pre VV	78
1 pre VF	61
1 sub VV	50
1 pre FF	47
1 pre FV	27
1 pre PP	27
1 pre PV	25
1 sub VF	23
1 sub FV	17
1 pre PF	15
1 sub FF	10
1 pre VP	8
1 sub PF	7
1 pre FP	5
1 sub PP	5
1 sub VP	4
1 sub PV	3
2 pre FV	3
2 preVF	2
2 pre VV	2
2 pre FF	1

The counts of the aggregated argument types are compiled in Table 4. As expected from the partial results, the proportion of *second-order arguments* is notably low: accounting for only 8 out of a total of 505 inference relations. Conversely, there is a high number of *default inference* classifications, especially when compared to the corresponding count for Walton’s taxonomy in Table 2. This label is assigned to any inference relation which could not be classified on at least one of the partial sub-classifications—an issue we return to in Sect. 5.2.

### Comparison of Results

The parallel annotation of the same original corpus with the two typologies of Sects. 3.1 and 3.2 opens up possibilities for comparative studies. In a qualitative sense, the dual annotation allows us to search for specific individual examples and analyse how these are respectively dealt with on a case-by-case basis. Quantitatively, the two resulting annotated corpora allow us to survey overall characteristics and draw more general conclusions.

In Sect. 4.1, we returned to Example (2) which was classified as *practical reasoning from analogy* in Walton’s classification. If we are interested in this particular type of argument, we can locate the same example in the US2016G1tvWAGEMANS corpus. On the basis of the PTA guidelines, it is classified as *1 pre PV*: a first order predicate argument with a value proposition as premise and policy proposal as conclusion. Such particular examples in the parallel corpora could be used to inform the comparison between the various types of argument scheme in each theoretical approach.

We can also compare the two annotations by cross-referencing the results numerically, as shown in Table 5. This co-occurrence matrix gives an overview of the correspondence between the overall results with the two typologies. To keep the table concise, we have excluded scheme classifications with fewer than three occurrences. This type of data is instrumental in, amongst others, the further development of the PTA by providing a basis for the specification of the ‘isotopes’ of the types of argument distinguished in the table (see Sect. 3.2.1). The co-occurrences show, for example, that arguments classified as *1 pre PF* according to the PTA—first order predicate arguments justifying a policy proposal on the basis of a factual proposition—tend to be associated with arguments from *consequences*, *example*, and three variations of *practical reasoning* and *pragmatic* argument schemes in Walton’s taxonomy.

**Table 5:** Co-occurrence matrix of argument schemes in US2016G1tvWALTON and US2016G1tvWAGEMANS

	Argument from alternatives	Argument from analogy	Argument from bias	Argument from cause to effect	Argument from composition	Argument from consequences	Argument from danger appeal	Argument from example	Argument from fear appeal	Argument from popular opinion	Argument from popular practice
1 pre FF	1	2	1	6	1	5	$\cdot$	13	$\cdot$	$\cdot$	1
1 pre FV	$\cdot$	$\cdot$	$\cdot$	2	$\cdot$	4	$\cdot$	4	$\cdot$	$\cdot$	1
1 pre FP	$\cdot$	$\cdot$	$\cdot$	$\cdot$	$\cdot$	$\cdot$	$\cdot$	3	$\cdot$	$\cdot$	$\cdot$
1 pre VF	$\cdot$	$\cdot$	1	11	1	6	$\cdot$	12	$\cdot$	$\cdot$	$\cdot$
1 pre VV	$\cdot$	1	2	9	1	2	1	13	$\cdot$	$\cdot$	$\cdot$
1 pre VP	$\cdot$	$\cdot$	$\cdot$	$\cdot$	$\cdot$	$\cdot$	$\cdot$	3	$\cdot$	$\cdot$	$\cdot$
1 pre PF	$\cdot$	$\cdot$	$\cdot$	$\cdot$	$\cdot$	2	$\cdot$	3	$\cdot$	$\cdot$	$\cdot$
1 pre PV	3	$\cdot$	1	$\cdot$	$\cdot$	3	$\cdot$	$\cdot$	$\cdot$	2	$\cdot$
1 pre PP	$\cdot$	$\cdot$	$\cdot$	$\cdot$	2	2	1	3	$\cdot$	$\cdot$	$\cdot$
1 sub FF	$\cdot$	2	1	$\cdot$	$\cdot$	1	$\cdot$	4	$\cdot$	1	$\cdot$
1 sub FV	2	1	$\cdot$	$\cdot$	$\cdot$	3	$\cdot$	$\cdot$	1	$\cdot$	2
1 sub VF	$\cdot$	2	$\cdot$	4	$\cdot$	1	$\cdot$	4	1	1	$\cdot$
1 sub VV	$\cdot$	$\cdot$	2	6	1	2	1	7	$\cdot$	1	$\cdot$
1 sub VP	$\cdot$	$\cdot$	$\cdot$	$\cdot$	$\cdot$	$\cdot$	$\cdot$	2	$\cdot$	$\cdot$	$\cdot$
1 sub PF	$\cdot$	$\cdot$	$\cdot$	$\cdot$	$\cdot$	1	$\cdot$	2	1	$\cdot$	$\cdot$
1 sub PP	$\cdot$	$\cdot$	$\cdot$	$\cdot$	$\cdot$	2	$\cdot$	1	2	$\cdot$	$\cdot$
Default Inference	3	$\cdot$	$\cdot$	10	$\cdot$	6	4	7	6	2	2

	Argument from position to know	Argument from sign	Argument from values	Argument from verbal classification	Circumstantial ad hominem	Ethotic argument	Generic ad hominem	Practical reasoning	Practical reasoning from analogy	Pragmatic argument from alternatives	Default Inference
1 pre FF	4	3	$\cdot$	2	$\cdot$	1	1	2	$\cdot$	1	2
1 pre FV	1	$\cdot$	$\cdot$	3	2	1	4	1	$\cdot$	$\cdot$	$\cdot$
1 pre FP	$\cdot$	$\cdot$	$\cdot$	$\cdot$	$\cdot$	$\cdot$	1	$\cdot$	$\cdot$	$\cdot$	$\cdot$
1 pre VF	2	13	$\cdot$	3	4	$\cdot$	4	1	$\cdot$	1	$\cdot$
1 pre VV	1	7	3	11	4	$\cdot$	6	9	$\cdot$	3	2
1 pre VP	$\cdot$	$\cdot$	2	1	$\cdot$	$\cdot$	$\cdot$	2	$\cdot$	$\cdot$	$\cdot$
1 pre PF	$\cdot$	$\cdot$	$\cdot$	$\cdot$	$\cdot$	$\cdot$	$\cdot$	2	2	3	$\cdot$
1 pre PV	1	$\cdot$	5	$\cdot$	$\cdot$	$\cdot$	$\cdot$	5	1	3	$\cdot$
1 pre PP	$\cdot$	$\cdot$	$\cdot$	1	$\cdot$	$\cdot$	$\cdot$	14	$\cdot$	4	$\cdot$
1 sub FF	1	$\cdot$	$\cdot$	$\cdot$	$\cdot$	$\cdot$	$\cdot$	$\cdot$	$\cdot$	$\cdot$	$\cdot$
1 sub FV	$\cdot$	$\cdot$	$\cdot$	1	2	1	2	1	$\cdot$	1	$\cdot$
1 sub VF	$\cdot$	2	$\cdot$	2	3	$\cdot$	$\cdot$	$\cdot$	$\cdot$	1	$\cdot$
1 sub VV	$\cdot$	4	3	4	8	$\cdot$	6	1	$\cdot$	$\cdot$	1
1 sub VP	$\cdot$	$\cdot$	1	$\cdot$	$\cdot$	$\cdot$	$\cdot$	1	$\cdot$	$\cdot$	$\cdot$
1 sub PF	$\cdot$	1	$\cdot$	$\cdot$	$\cdot$	$\cdot$	$\cdot$	$\cdot$	$\cdot$	1	$\cdot$
1 sub PP	$\cdot$	$\cdot$	$\cdot$	$\cdot$	$\cdot$	$\cdot$	$\cdot$	$\cdot$	$\cdot$	$\cdot$	$\cdot$
Default Inference	2	7	1	2	1	2	3	6	1	4	12

Of the 505 arguments in the original US2016G1tv corpus, most can be classified under both annotations: on the basis of Walton’s taxonomy, 97% is classified as something other than *default inference*, which drops to 83% for annotation with the PTA. There are 12 instances that defy classification under either approach. Several factors conspire to result in these elusive cases. Firstly, the argumentation structures in US2016G1tv were not initially annotated with scheme classification in mind. The resulting structures can thus be difficult to match to the specification constraints in both Walton’s taxonomy and Wagemans’ Periodic Table of Arguments. Additionally, some of the propositions annotated as inferentially connected by the original IAT annotators of the US2016G1tv corpus turn out not to be amenable at all to classification of the underlying argument scheme. This can be the result of annotation mistakes in US2016G1tv, where something may mistakenly have been annotated as an argumentative inference relation while none is present—perhaps by misinterpreting a causal discourse marker as reliable indicator of argumentative inference, such as ‘so’ in Example (3). In other cases, an utterance may justifiably be interpreted as expressing an intention to draw an inference—duly annotated as such in the pre-existing IAT annotation of US2016G1tv—while it is not clear at all what type of scheme is used, such as in Example (4).

(3): Donald Trump: *We’re just opening up on Pennsylvania Avenue right next to the White House, so if I don’t get there one way, I’m going to get to Pennsylvania Avenue another.*
(4): Donald Trump: *Sean was in favour of the war. And I understand that side, also, not very much, because we should have never been there.*

A notable difference between the annotations with Walton’s taxonomy and with Wagemans’ PTA is that the latter produces intermediate classification results in addition to overall results. This is the case even if the final classification fails. In particular, the annotation of proposition types is relevant and beneficial to the verification of premise acceptability (Freeman 2000), and the persuasiveness of an argument (Reynolds and Reynolds 2002; Hoeken and Hustinx 2003). Both can only be reliably appraised when knowing against what standard this should be done—whether it be correctness of stated facts, agreement with expressed values, or adequacy of proposed policies. Table 3 shows the results of the classification of the 798 propositions in the corpus: the majority of 376 is classified as *value*, followed by 298 propositions of *fact*, and 108 propositions of *policy*—with a Cohen’s $\kappa$ (Cohen 1960) of 0.778 (see Sect. 5.2). A disadvantage of breaking down the annotation task into constitutive sub-classifications is that each stage risks introducing obstacles for the final classification. If in any of three associated sub-tasks it is not entirely clear how to classify one part of an argument, then the overall final classification will end up as *default inference*. In Sect. 5.2 we address the resulting relatively high number of *default inference*s in the PTA annotation, and suggest a solution.

## Evaluation and Revision of Annotation Methods

### Discussion of Guidelines for Walton’s Taxonomy

The annotation of argument schemes on the basis of Walton’s taxonomy is evaluated by calculating the agreement between the two annotators. For this purpose, a 10.2% random sample of all inference relations in the US2016G1tv corpus was annotated by both annotators. This yields a Cohen’s $\kappa$ (Cohen 1960) of 0.723—well within substantial agreement according to the Landis and Koch (1977) interpretation.

Some classes of argument scheme turned out to be particularly difficult to distinguish. For example, Example (5) was classified by one annotator as *practical reasoning*, related to promoting goals, and by the other as *argument from values*, related to promoting values. This difficulty is also reflected in the fact that Walton et al. (Walton et al. 2008, p. 324) describe a subtype of *practical reasoning* called *value-based practical reasoning*—itself not included in our annotation guidelines, because of the restriction to top-level argument schemes.

(5): Hillary Clinton: *What I have proposed would be paid for by raising taxes on the wealthy [...] I think it’s time that the wealthy and corporations paid their fair share to support this country.*

The annotated corpus contains 14 *Default Inference* classifications. There are two explanations for these arguments defying classification on the basis of the guidelines of Sect. 3.1.2. As we discussed in Sect. 3.1.1, Walton’s taxonomy of argumentation schemes should not be considered as fixed, but rather as an developing system. So while the current annotation guidelines cover all 60 main scheme types, this list should not be taken as comprehensive—neither in terms of exhaustively describing all possible types of argument, nor of detailing all ways of distinguishing between similar types. Additionally, the original IAT annotation of argument structure in the US2016G1tv corpus was not done with argument schemes in mind. Some constellations of premises and conclusions would hence be structurally annotated in a different way if the objective of matching them to Walton’s argumentation schemes was taken into account from the start.

In comparison to other annotation methods for argument schemes (Sect. 2.2, our method of supporting the annotation with a heuristic decision tree (Sect. 3.1.2) leads to reliable inter-annotator agreement and a wide variety of identified scheme types. For this reason, we further developed this heuristic method, from its first incarnation to a robust annotation tool that could support trained experts, and non-experts alike. To this avail, we recognise that the annotation procedure relies heavily on the distinctive properties of arguments that are characteristic for a particular scheme. In that sense, the procedure bears a striking resemblance to biological taxonomy, the identification of organisms in the various sub-fields of biology (see, e.g., Voss 1952; Pankhurst 1978).

Drawing on this biological analogue, we envision a taxonomic key for argument scheme annotation. A taxonomic key can be seen as a textual rendering of a decision tree—serving the same function. For identifying biological organisms, keys have long proven suitable for both experts and the masses, without the need for specialist training—which becomes relevant when considering the prospects of crowd-sourcing the annotation task (Musi et al. 2016).

In “Appendix A” we specify a taxonomic key for the identification of argument schemes in accordance with Walton’s taxonomy: the Argument Scheme Key (ASK), version 1.^4^ The ASK is a dichotomous identification key that leads the analyst through a series of disjunctive choices based on the distinctive features of a ‘species’ of argument scheme to the particular type. The choices are informed by grouping together scheme types in Walton’s taxonomy that share particular characteristics. For example, the ASK starts by distinguishing between source-based and other arguments. Each subsequent choice in the key leads to either a particular argument scheme, or to another numbered entry with a further distinction. The entries with distinctive characteristics are numbered—following the standard in Biology—with between brackets the number of the previous characteristic that led to this particular point in the key.

For example, an analyst goes through the following sequence of numbered characteristics in identifying an argument as an instance of *argument from popular opinion*:

- 1 source-based;
- 2 about the source’s opinion;
- 9(2) based on existing opinion;
- 11(9) source is a group of people.

### Discussion of Guidelines for Wagemans’ Periodic Table of Arguments

The PTA annotation is validated by calculating the inter-annotator agreement for the three partial classifications, as well as for the final aggregated schemes. For the classification of propositions as statements of fact, value, or policy, a random sample of 13.4% was annotated by both annotators, resulting in a Cohen’s (1960) $\kappa$ of 0.778. The classification of predicate arguments and subject arguments yields a Cohen’s $\kappa$ of 0.851 on a 10.0% sample. Also on a 10.0% sample, the classification of first-order arguments and second-order arguments results in a Cohen’s $\kappa$ of 0.658. The inter-annotator agreement for the overall aggregated scheme classification on the basis of the PTA results in a Cohen’s $\kappa$ of 0.689 on a 10.4% sample.

The agreement scores on the partial and final PTA annotations all fall within the range of substantial to almost perfect agreement (Landis and Koch 1977), suggesting that the division into independent sub-tasks simplifies the annotation while maintaining reliability. Consequently, we envision that this type of annotation with the PTA could lend itself well to a crowd-sourced or otherwise untrained/non-expert annotation approach—amongst others, Miller et al. (2019) show that decomposing complex argument annotation tasks into simpler constitutive sub-tasks makes them suitable for crowd annotation.

A comparison between the agreement scores for the three PTA annotation sub-tasks shows that the classification of arguments as first-order or second-order leads to the least reliable results—while the $\kappa$ of 0.658 still amounts to substantial agreement (Landis and Koch 1977). We hypothesise that the lower score is a result of the unbalanced nature of the dataset, with a strong predominance of first-order arguments. As the compiled partial results in Table 3 show, the second-order arguments account for only 11 out of the total of 505 inference relations, compared to 481 first-order arguments. This imbalance has a strong impact on the Cohen’s $\kappa$ metric, which becomes all the clearer upon calculating the corresponding percentage-agreement: the two annotators agreed in their first-/second-order classification in 98.0% of the cases.

The most common aggregate type in the US2016G1tvWAGEMANS corpus is *Default Inference* at 17%—see Table 4. This high proportion results from the aggregation of the partial results into the final argument types. Because the three partial results are combined into the final types, the constitutive propositions and inferential relation all have to be classified individually in the sub-tasks. Therefore, a failure to classify any of the individual components will cause the overall argument type to default into the *Default Inference* category. If a proposition cannot be classified in terms of policy/value/fact (for example, because it is too vague), or if the relation is not clearly first- or second-order, or if the propositions are incomplete to the extent that it is not clear whether the subject or predicate is responsible for the transferring of justificatory force, then the aggregated final classification of the argument as a whole fails, resulting in a *Default Inference*.

The structural characteristics of the IAT annotation complicate matters for subsequent PTA annotation. IAT caters for arguments with multiple premises, and complex structures of argumentation (such as linked arguments or coordinatively compound argumentation). In contrast, the PTA only considers arguments consisting of one proposition as premise, and one proposition as conclusion.

Another complication stems from the source material on which the annotation of schemes builds. As a result of, for example, interruptions, corrections, and general obscurity, the speech in the transcribed election debates is incomplete or not syntactically well-formed. The PTA, in contrast, presupposes that premises and conclusions of arguments consist of complete categorical propositions comprising a clear subject-predicate structure. However, the preexisting IAT annotation of the original US2016G1tv corpus does not reconstruct the transcribed text of the election debate to that degree of detail. Since the starting point of the annotation with the PTA was that the original structural IAT annotation would be left unchanged, an interpretative step is required to get from the naturally expressed argument in the data to a proposition that can be classified. This means that it is up to the analysts to correctly interpret the propositions while doing the PTA classifications—something that can be difficult, as becomes clear from an example such as (6), comprising two propositions connected with the discourse marker ‘because’, indicative of argumentation (van Eemeren et al. 2007).

(6): Donald Trump: *And one of your compatriots said, you know, whether it was before or right after, Trump was definitely—because if you read his article, there’s no doubt.*

Table 3 shows that all but 13 inference relations could be classified as *first-* or *second-order*, and all but 14 propositions as *value*, *policy* or *fact*. Classification as *subject* or *predicate* argument, however, failed in 73 cases. This led Visser and Wagemans (2018) to propose a revision of the annotation guidelines by operationalising the notion ‘argument form’ in accordance with the most recent iteration of the theoretical framework of the PTA (Wagemans 2019a, b). The argument form can be determined by considering up to three heuristic questions (as shown in the decision tree reproduced in “Appendix B”), resulting in a combination of the first two partial characterisations of arguments—as a *first-order* or *second-order* argument and as a *subject* or *predicate* argument. The combination of the two characteristics yields four possible argument forms, constituting the four quadrants of the PTA:

- *first-order predicate arguments*, instantiating the form ‘a is X, because a is Y’, and constituting the Alpha Quadrant,
- *first-order subject arguments*, instantiating the form ‘a is X, because b is X’, constituting the Beta Quadrant,
- *second-order subject arguments*, instantiating the form ‘q is T, because r is T’, constituting the Gamma Quadrant,
- and *second-order predicate arguments*, instantiating the form ‘q is T, because q is Z’, constituting the Delta Quadrant.

Given that the guidelines used for the present annotation (see Sect. 3.2.2 ) only mention instructions for identifying second-order predicate arguments, one would expect the relatively high number of default inferences to drop if the annotation is carried out by using the revised guidelines that include instructions for identifying second-order subject arguments as well.

A final problem we will discuss here is related to the third characteristic mentioned in the theoretical framework of the PTA—the specific combination of statement types substantiated by the conclusion and the premise of the argument. As explained in Sect. 3.2.1, the PTA distinguishes between three types: statements of *value*, *policy*, and *fact*. The annotators reported that it was sometimes particularly difficult to distinguish statements of *value* from the other two types—especially from statements of *fact*. For example, Trump’s statement (7), on the topic of Clinton’s use of a private e-mail server, was classified by one annotator as a statement of *fact*, and by the other as a statement of *value*. The question here is whether the emphasis is on the evaluative judgment about the legality of the act, or rather on the factual description of someone invoking the Fifth Amendment of the US Constitution to refuse testimony.

(7): Donald Trump: *Clinton has the man that set up the illegal server taking the Fifth Amendment*

This kind of qualitative evaluation of the annotation guidelines indicates the need for a more elaborate description of what counts as a specific type of statement. Complementing the notion ‘argument form’, Visser and Wagemans (2018) reframe the third partial characteristic as ‘argument substance’. Additionally, they propose to revise the description of statements of value as statements expressing an evaluative judgment about something that is based on a subjective selection and weighing of assessment criteria. To help the analyst distinguish statements of *value* from statements of *fact*, the following examples of sub-types provide guidance in determining the ‘substance’ of an argument:

- *Statements of value:*    A statement expressing an evaluative judgment about something that is based on a subjective selection and weighing of assessment criteria. The following examples of sub-types should help the analyst distinguish them from statements of fact:
  - aesthetic judgments, such as ‘*The Corrections* is a great novel’
  - moral or ethical judgments, such as ‘Circumcision is reprehensible’
  - legal judgments, such as ‘Unauthorized copying is not theft’
  - pragmatic judgments, such as ‘Our plan for reducing CO2-emission is feasible’
  - logical judgments, such as ‘This proposition is true’
  - hedonistic judgments, such as ‘Paragliding is fun’.

Together, the operationalisation of Visser and Wagemans’ (2018) notions of ‘argument form’ and ‘argument substance’ constitute revised annotation guidelines in the form of an *Argument Type Identification Procedure* (ATIP).^5^

## Applications of Annotated Argument Scheme Corpora

### Argument Mining

Argument mining (Stede and Schneider 2018; Lawrence and Reed 2019) is a rapidly growing field, with an increasing number of methods being developed to automatically process textual data and reconstruct argumentative content. However, most of these techniques are limited by the lack of consistently annotated argument data at sufficiently large scale. The use of crowdsourced annotation (Ghosh et al. 2014; Skeppstedt et al. 2018) and automatic methods to extend the data currently annotated (Bilu et al. 2015) have helped this situation somewhat, though even these struggle with more fine grained annotation such as the annotation of argument scheme instances.

One of the first approaches to mining Walton’s argument schemes (Feng and Hirst 2011), uses the Araucaria corpus (Reed 2006) as training and test data for a machine learning classifier that aims to distinguish between instances of the top five most commonly occuring schemes in this corpus. Whilst the results for this task are promising (with accuracies of 0.63–0.91 achieved in one-against-others classification and 0.80–0.94 in pairwise classification) the small number of scheme instances and lack of explicit, validated annotation guidelines means that this dataset cannot be used to explore a full range of scheme types.

More recent techniques for mining schemes have either used small sets of hand curated scheme examples (Lawrence and Reed 2016) or developed specific classifications to aid the creation of larger datasets in varying domains—for example: Green (2015) lists ten custom argument schemes targeted at genetics research articles; Wyner et al. (2012) describe a consumer argument scheme, with the structure of this scheme used to guide an argument identification process.

Walton (2011) also notes the lack of a systematic approach to computationally identifying arguments and their schemes. To address this challenge, we might first identify the arguments occurring in a piece of text, followed by the identification of specific known argument schemes. Beyond this initial identification, however, there are likely to be issues differentiating between similar schemes, which can be addressed by developing a corpus of borderline cases.

With the data currently available, the ontologically rich information provided by argument schemes has been demonstrated to be a powerful component of a robust approach to argument mining. Collaboration amongst analysts as well as the further development of tools supporting schemes (such as the OVA online annotation tool (Lawrence et al. 2019)) is essential to growing the datasets required to improve on these techniques. Clear annotation guidelines for Walton’s taxonomy of schemes, such as the Argument Scheme Key (see Sect. 5.1 and “Appendix A”), will hopefully result in a rapid growth in the material available and further increase the effectiveness of automated schematic classification.

The sub-task of classifying proposition types in accordance with Wagemans’ Periodic Table of Arguments (PTA) typology (Sect. 3.2) also resonates with existing work in argument mining. Park and Cardie (2014) distinguish between three proposition types (unverifiable, verifiable non-experiential, and verifiable experiential), aiming to automatically identify each of these in online user comments in order to highlight propositions which are insufficiently supported. In subsequent work (Park and Cardie 2018), the authors revise their typology to include the three proposition types of the PTA: propositions of non-experiential fact (*fact*); propositions of value (*value*); propositions of policy (*policy*); propositions of experiential fact (*testimony*); and reference to a resource (*reference*). Others have also followed similar proposition type classifications: Dusmanu et al. (2017) distinguish between factual and opinion-based arguments on Twitter; while Al Khatib et al. (2016) classify argumentation strategies in terms of *common ground*, *assumptions*, *testimony*, *statistics*, *anecdotes*, and *other*; and Rinott et al. (2015) distinguish between three evidence types: *study*, *expert*, *anecdotal*.

The classification of propositions as factual in particular has gained prominence as part of fact-checking endeavours in combating fake news. Hassan et al. (2015), for example, classify sentences as *non-factual*, *unimportant factual*, and *check-worthy factual*. Similarly, Patwari et al. (2017) and Jaradat et al. (2018) automatically determine the ‘fact-check-worthiness’ of factual claims in political debates. Naderi and Hirst (2018) automatically distinguish between *true*, *false*, *stretch*, and *dodge* statements in parliamentary proceedings.

### Rhetorical Profiling

The availability of argumentatively annotated text corpora of appropriate size and quality opens up new possibilities for applying quantitative empirical methods in the study of argumentation. On the basis of the corpora we present in the current paper, we can explore the use of corpus-based metrics for Argument Analytics. Introduced by Lawrence et al. (2016), Argument Analytics provide a suite of automated techniques for statistical analysis and infographics-style visualisation to produce intuitive insights into large-scale argumentative discourses. Extending the existing methods, we can construct an empirically-grounded *rhetorical profile* of a speaker, by matching the classification of arguments with who advanced them. This allows us to characterise the speakers’ style of arguing in terms of, for example, their selection of argument schemes, and the type of standpoints they advance.

We employ these data-driven characterisations to rhetorically profile the relative styles of Clinton and Trump in their first head-to-head television debate (see Sect. 2.3.1). Combining aspects of the original IAT-based annotation with the annotations with the Waltonian argumentation schemes (Sect. 3.1) and the Periodic Table of Arguments (Sect. 3.2), the corpus-based analytics allow us to show differences in the rhetorical styles of the two candidates on the basis of quantitative empirical evidence.

During the debate, Trump spoke for 45min3sec, and Clinton for 41min50sec. In their respective speaking time, Trump advanced 292 arguments, while Clinton accumulated a much lower total of 194. As expected in political debates, both Clinton and Trump regularly made use of *Arguments from Example*, *Cause to Effect*, *Sign*, and *Consequences*. Striking is Trump’s propensity for personal attacks, such as in Example (8). 15% of his arguments consist of *Circumstantial* or *Generic Ad Hominem* and *Argument from Bias*, compared to 7% of Clinton’s.

(8): Donald Trump: *And she doesn’t say that, because she’s got no business ability. [...] But you have to have some basic ability. And sadly, she doesn’t have that. All of the things that she’s talking about could have been taken care of during the last 10 years, let’s say, while she had great power.*

Trump also uses a considerably higher number of *Fear Appeals* to justify his standpoints: 10 for Trump (making up 3.4% of his total number of arguments), against 1 for Clinton (0.5%). Clinton, on the other hand, relies more heavily on *Popular Opinion* and *Popular Practice* argument schemes than Trump does: 10 counts for Clinton (constituting 5% of her arguments) against 4 counts for Trump (1%). Furthermore, she employs the *Argument from Values* 10 times (5.2% of her arguments), while Trump only relies on values 5 times (1.7%).

Another stark difference in the rhetorical choices made by Clinton and Trump is the type of claims defended. In political debates, especially in election times, we might expect to find a high proportion of policy proposals—such as the one in Example (2). Indeed, in 28% of the cases, Clinton argues in defence of a standpoint constituting a statement of policy. Trump however only support statements of policy in 9% of his arguments. This distinctive difference in rhetorical style is further confirmed by the candidates’ use of the *Practical Reasoning* argument scheme, in which a plan of action is defended on the basis of a particular goal: 17% of Clinton’s arguments constitute *Practical Reasoning*, against 4% of Trump’s.

## Conclusion

Adopting a corpus-linguistic approach to argument schemes, we introduce, apply, and revise practical guidelines for the annotation of corpora of real-world argumentative data. Consequently, we present a text corpus annotated on the basis of two distinct typologies of schemes: Walton’s taxonomy of argumentation schemes and Wagemans’ Periodic Table of Arguments (PTA). The two resulting annotated corpora should prove useful both for quantitative empirical approaches to the study of argumentation, and to computational research into argument mining—the automated reconstruction of argumentative content in natural language texts of arbitrary length (Stede and Schneider 2018; Lawrence and Reed 2019).

For each of the two classifications of schemes, we describe and validate the annotation procedure, and present the resulting annotated text corpora (Sects. 3.1 and 3.2). In doing so, we extend the annotation of the pre-existing US2016G1tv corpus, comprising the first television debate between Hillary Clinton and Donald Trump for the 2016 US presidential elections (Visser et al. 2019a) (Sect. 2.3.1). To the best of our knowledge, the resulting two corpora are the largest of their kind: publicly available corpora of argumentation annotated with discourse structure, speech acts, argument structure, and two versions of scheme types. Based on an evaluation in terms of Cohen’s $\kappa$ (Cohen 1960) yielding at least substantial inter-annotator agreement across the board, we suggest revisions to the guidelines for annotating schemes: the Argument Scheme Key (ASK) for annotation with Walton’s taxonomy (“Appendix A)”, and the incorporation of a distinction between *argument form* and *argument substance* in the Argument Type Identification Procedure (ATIP) for annotation with Wagemans’ PTA (“Appendix B”). The presented methods should provide solid foundations for the development of robust and diverse datasets for the empirical study of argumentation, and for applications in AI and machine learning alike.

The parallel annotation of the same US2016G1tv corpus on the basis of both Walton’s taxonomy and Wagemans’ PTA opens up new avenues for quantitative research into argumentation. While comparing and reconciling different approaches to scheme classification is not one of our objectives in the current paper, the methods presented and data obtained are useful for such purposes. Neither of the two approaches we discuss should be considered complete or final, while both start from very different theoretical foundations. Walton’s taxonomy comprises a great many schemes described in varying detail. It should not be regarded as a completed structure, but as a work in progress that is continually readjusted and refined as the concepts defining the schemes are formulated in a more precise way and applied to new examples. Similarly, Wagemans’ PTA is under constant development. His approach, however, is based on an a priori constrained set of possible combinations between three distinct characterisations of argument, used to scaffold the systematisation of encountered instances of argument types. While Walton’s approach provides the flexibility and richness needed to apply the general methodology to the particular needs of a research project or practical application, Wagemans’ approach provides an exhaustive set of possible classifications, much needed in computational applications. Comparative data (such as the co-occurrence matrix of Table 5) can contribute directly to any attempts of crossing the foundational starting points of the different typologies—for purposes of both reconciliation and contrasting.

While our annotation approach results in the largest and most reliable datasets of their kind, the fact that we also propose possible ways of improving the annotation guidelines shows that the work we present here is not without its limitations. The original US2016G1tv corpus, for example, was not specifically developed with future scheme annotation in mind. The result is that some of Visser et al. (2019a)’s structural annotations do not map nicely to the technical scheme specifications in either of the typologies we consider. Furthermore, the corpus is constrained to one election debate, and hence to a constrained set of speakers communicating in one particular genre. It is conceivable—although not likely—that the annotation procedures we describe are somehow not generalisable beyond these speakers or this genre, even though the guidelines are in no way contextually tailored. In previous work, Walton’s taxonomy has been used to annotate corpora from different contexts, but results have varied (see Sect. 2.2). With respect to the PTA, the annotation we present here constitutes a first, so there is nothing to compare to. Lastly, the annotations reported on in Sect. 4 and evaluated in 5 are obtained by using only two experienced annotators. We suggest future annotation studies can experiment with multiple annotators and varying levels of experience, using texts from diverse genres with dedicated argumentation-structural annotation, to verify the extend to which our methods and suggested revisions are generally applicable.

## Figures and Tables

**Fig. 4:**
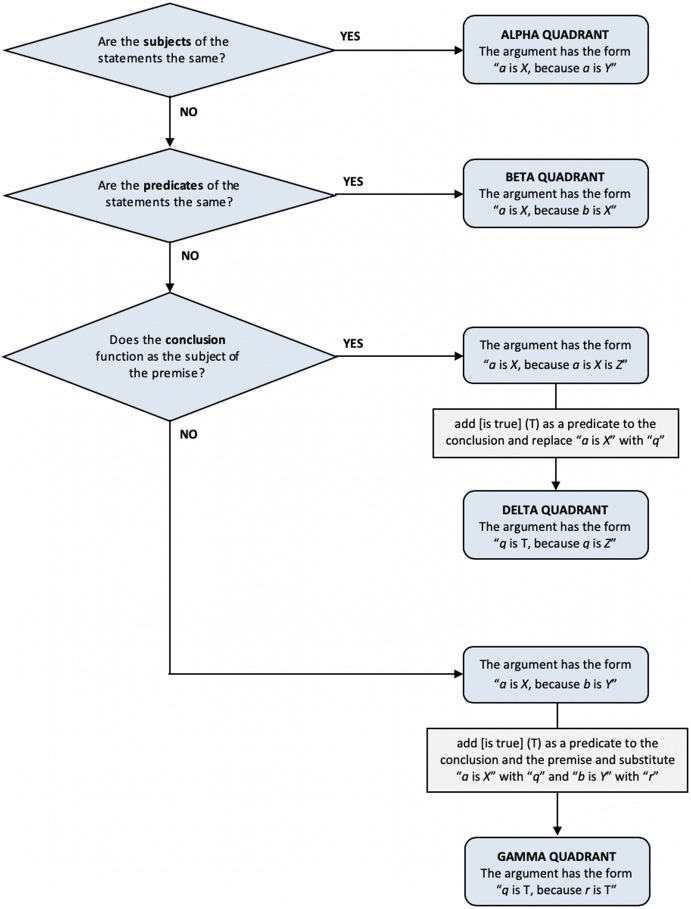
Decision tree for determining argument form in the Argument Type Identification Procedure (ATIP)

## Appendix B: ATIP Decision Tree for Determining Argument Form

See Fig. 4.

## Footnotes

1 In the literature, various authors use different terms to signify the same general idea (with small variations): e.g., ‘argument scheme’ (van Eemeren and Grootendorst 1992), ‘argumentation scheme’ (Walton 1996), and ‘argumentative scheme’ (Perelman and Olbrechts-Tyteca 1969). In the present paper, we will predominantly use the term ‘argument scheme’.

2 Not all current accounts of argument(ation) schemes emphasise this communicative angle, favouring a reinterpretation of the interactional critical questions as static exceptions or defeaters (van Eemeren et al. 2014).

3 Depending on the needs or the requirements of the application, the categorisation can be further differentiated by adding another partial characteristic as a constituent to the theoretical framework. For present purposes, we focus on the three standard constituents of *first*-/*second* *order*, *subject*/*predicate*, and propositions of *value*/*policy*/*fact*.

4 In the ASK, we include the *argument from consequences* twice, in its positive (marked +) and its negative (marked –) variant. We decided to include both because the characteristics that allow annotators to distinguish these schemes lead to two very different routes through the identification key.

5 The ATIP (Wagemans 2019a) is available in full online at http://www.periodic-table-of-arguments.org/argument-type-identification-procedure.
